# Supplementary material for: Mesenchymal Stromal Cells Regulate Sialylations of N-Glycans, Affecting Cell Migration and Survival
Source: Int J Mol Sci. 2021 Jun 26;22(13):6868. doi: 10.3390/ijms22136868 (PMC8267656; doi:10.3390/ijms22136868)
Supplement: Supplementary file 1 [file ijms-22-06868-s001.zip › ijms-1247574-supplementary.pdf]

International Journal of *Molecular Sciences*

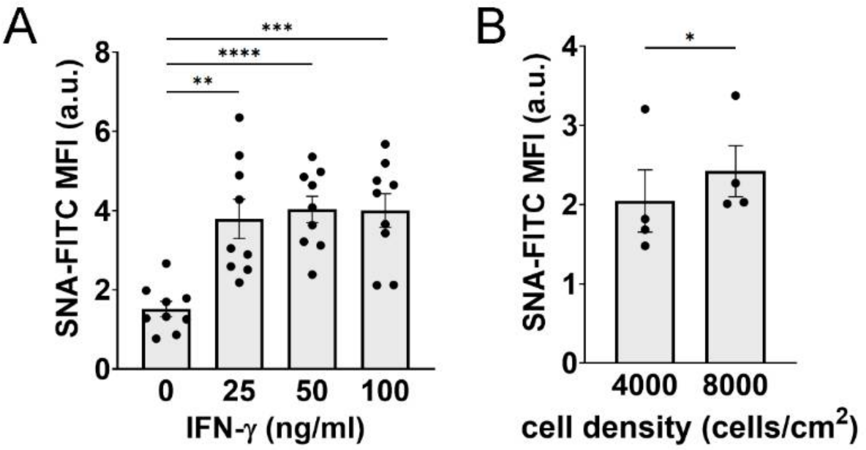

Supplementary Figure S1. IFN-γ and high density increase sialylations of N-glycans in MSCs. (A) MSCs were cultured for 24 hours with various concentrations of IFN-γ and analyzed by flow cytometry to determine sialylations by means of SNA binding (n = 9). (B) MSCs were cultured for 48 hours at the indicated cell densities prior to flow cytometry analysis using SNA staining (n = 4). * $p < 0.05$; ** $p < 0.005$; *** $p < 0.0005$; **** $p < 0.00005$.

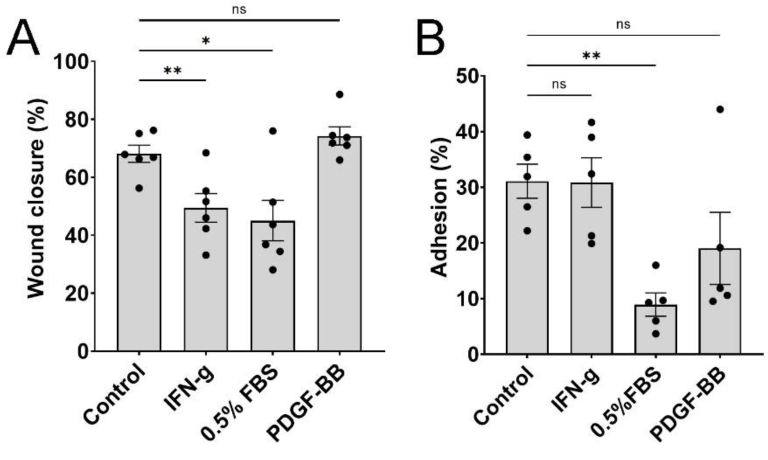

Supplementary Figure S2. Effect of IFN-γ, 0.5% FBS and PDGF-BB on migration and adhesion of MSCs. (A) MSCs pre-treated with the respective conditions were tested in wound/scratch assays to determine effects on cell migration. Here, pre-treatment with IFN-γ or with 0.5% FBS (instead of 10% FBS, Control) significantly reduced wound closure, while PDGF-BB had no significant impact. (B) Adhesion assays of MSCs pre-treated as in A. Here, only 0.5% FBS significantly affected cell adhesion by reducing it. ns: not significant; * $p < 0.05$; ** $p < 0.005$, as determined using 1-way ANOVA and post hoc Tukey test.

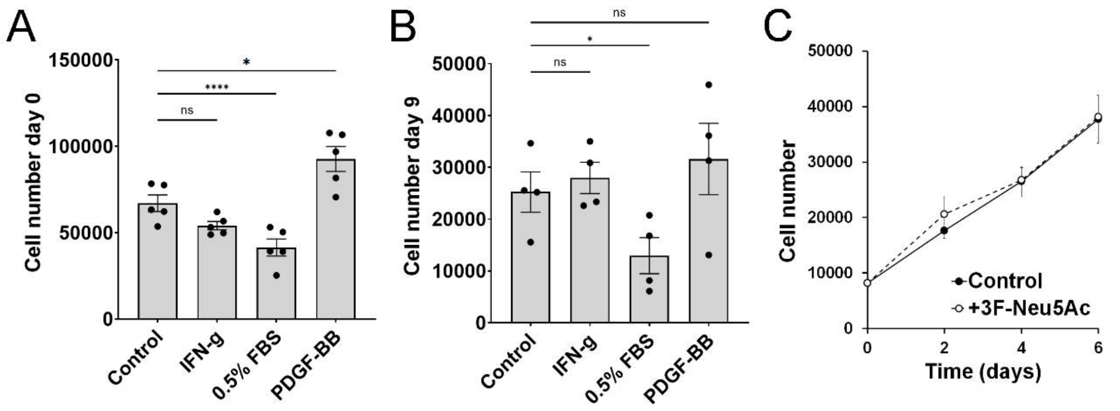

Supplementary Figure S3. Effect of IFN-γ, 0.5% FBS, and PDGF-BB on proliferation and survival of MSCs and effect of 3F-Neu5Ac on cell proliferation. (A) Cells were pre-treated for 48 hours under the indicated conditions. Then (time 0), cells were lifted and counted (n = 5). (B) Cells were treated as in A, but at time 0, medium was changed to serum-free media and cells were transferred to an hypoxic incubator (1% Oxygen), without further medium changes. After 9 days, the remaining cells were lifted and counted (n = 4). (C) Cell proliferation assay of cells treated with or without 3F-Neu5Ac (50μM). At each time point, cells (plated in triplicate) were lifted and counted using hemocytometer and trypan blue exclusion dye (n = 4). ns: not significant; * $p < 0.05$; **** $p < 0.00005$.
